# Local understandings of care during delivery and postnatal period to inform home based package of newborn care interventions in rural Ethiopia: a qualitative study

**DOI:** 10.1186/1472-698X-14-17

**Published:** 2014-05-19

**Authors:** Tedbabe Degefie, Yared Amare, Brian Mulligan

**Affiliations:** 1 Newborn and Child Health Consultant, Silver Spring MD, USA; 2 Consultancy for Social Development, Addis Ababa, Ethiopia; 3 JSI Research and Training Institute Inc, Yangon, Myanmar

**Keywords:** Ethiopia, Newborn care, Qualitative methods, Cultural beliefs

## Abstract

**Background:**

Despite a substantial decrease in child mortality in Ethiopia over the past decade, neonatal mortality remains unchanged (37/1000 live-births). This paper describes a qualitative study on beliefs and practices on immediate newborn and postnatal care in four rural communities of Ethiopia conducted to inform development of a package of community-based interventions targeting newborns.

**Methods:**

The study team conducted eight key informant interviews (KII) with grandmothers, 27 in-depth interviews (IDI) with mothers; seven IDI with traditional birth attendants (TBA) and 15IDI with fathers, from four purposively selected communities located in Sidama Zone of Southern Nationalities, Nations, and Peoples (SNNP) Region and in East Shewa and West Arsi Zones of Oromia Region.

**Results:**

In the study communities deliveries occurred at home. After cutting the umbilical cord, the baby is put to the side of the mother, not uncommonly with no cloth covering. This is largely due to attendants focusing on delivery of the placenta which is reinforced by the belief that the placenta is the ‘house’ or ‘blanket’ of the baby and that any “harm” caused to the placenta will transfer to the newborn. Applying butter or ointment to the cord “to speed drying” is common practice. Initiation of breastfeeding is often delayed and women commonly report discarding colostrum before initiating breastfeeding. Sub-optimal breastfeeding practices continue, due to perceived inadequate maternal nutrition and breast milk often leading to the provision of herbal drinks. Poor thermal care is also demonstrated through lack of continued skin-to-skin contact, exposure of newborns to smoke, frequent bathing—often with cold water baths for low-birth weight or small babies; and, poor hygienic practices are reported, particularly hand washing prior to contact with the newborn.

**Conclusion:**

Cultural beliefs and newborn care practices do not conform to recommended standards. Local perspectives related to newborn care practices should inform behaviour change messages. Such messages should target mothers, grandmothers, TBAs, other female family members and fathers.

## Background

Globally, the progress toward reducing newborn mortality has been slow [1,2]. Despite the fact that Ethiopia has made substantial improvement in child mortality; neonatal death accounts for near half (42%) of all under-5 child deaths. Annually, more than 110,000 newborns die in Ethiopia, with the greatest numbers in rural areas [3]. The risk of death is greatest in the first day of life, when half of neonatal deaths occur, and some three-quarters of deaths occur within the first week of life, the early neonatal period. Birth asphyxia (intrapartum-related complications) and sepsis account for near half of deaths, followed by preterm birth complications globally and in Ethiopia [4-7].

Both The Lancet series on Newborn [8] and on Maternal [9] Health suggest that 15 to 32% of neonatal deaths can be prevented through achieving high coverage of a few key practices: clean home delivery, hygienic cord care, thermal care, early and exclusive breastfeeding, community-based care for low birth weight and care seeking for illness in low income countries. The recommended interventions emphasize strengthening the continuum of maternal, newborn and childcare including antenatal care (ANC), intra-partum care and postnatal care (PNC) for the mother and the newborn [10-14]. Trained community workers are considered by many to be pivotal for improved newborn care in the community [14,15], and studies have shown results that they could have a significant impact on neonatal mortality and uptake of key behaviours and practices [16-18].

Most of these recommendations are based on studies conducted in Asia [19-21]. There are only a few studies from Sub-Saharan Africa (SSA) on community beliefs and practices that influence maternal and newborn health [22,23].

Understanding such beliefs and practices that promote or hinder health and survival is central to developing strategies to ensure positive outcomes for both the mother and baby [24,25]. For example a study in Uganda showed strong care takers beliefs that new-borns are born dirty and smell drove harmful practices of immediate bathing. In the same line the belief that application of different substances to cord help it heal fast and the seclusion is needed until the cord falls hindering postnatal care [22]. Waren reported initiation of breast feeding delayed up to three days because of a belief that colostrum is unhealthy for the baby and first option of care is traditional healer as illness are caused by “evil eye” [23]. We conducted this study to answer the following questions: 1. what are the local rationales and perceptions behind local delivery and postnatal care practices? 2) who participates in or influences newborn care? 3) what are the implications of practices and perspectives related to local delivery and postnatal care for behavioral changes messages?

## Methods

Qualitative research methods employed consisted of key informant interviews and in-depth interviews conducted in four *kebeles* (villages) purposively selected to represent varying proximity to health facilities. One *kebele* was selected in one of the following *woredas* (districts): AletaChuko and Arbe Gonna in Sidama Zone in SNNPR Region, and Liben Chiquala district in East Shewa Zone and Gedeb Asasa district in West Arsi Zone of Oromia Region.

The population represented by the study communities consists predominantly of rural farmers who also maintain livestock. Sidama Zone is populated by the Sidama people, one of more than 40 ethnic groups in SNNPR. They are mostly Protestant Christians. East Shewa Zone is located in Central Oromia and is populated by ShewaOromos. They are predominantly Orthodox Christians although participants in the study appear to be mostly Protestant Christians. West Arsi Zone is located south of East Shewa and is populated by Arsi Oromo who are Muslims. The study was conducted from June to October, 2012. Community facilitators selected mothers who had delivered less than three months before the interview and varied on the basis of parity. Fathers and grandmothers were also usually selected from the same households as the mothers. One trained and one untrained TBA was selected from the few TBAs available in each community.

The study team conducted a total of eight key informant interviews with grandmothers, twenty seven IDIs with mothers, seven IDI with TBAs, fifteen IDIs with fathers. Mothers’ age range 18 to 35 years. All are married and from Sidama ethnic group and Protestant Christians in SNNPR, Oromo and Orthodox or Protestant Christians in East Shoa, and Oromo and Muslim in West Arsi. Ethnic differences coincide with different study communities. Mothers were selected purposively on the basis of having a baby in the last three months before the interview and variation by parity. Although the small number of babies delivered in the last three months in a village limited the direct use of age and education as sampling criteria, the final sample exhibited some variation in these variables. Grandmothers and TBAs were predominantly older and illiterate, whereas fathers were selected from the same households as the mothers.

The interview tools were designed to address the research questions outlined above “see Additional file 1”. Local rationales and perspectives related to care practices were investigated by inquiring into the nature of and reasons for engaging in such practices and beliefs regarding factors behind health outcomes. Inquiries were also made about who participates in and provides advice regarding various maternal and newborn care practices. Findings from such inquiries were expected to yield insights regarding the design of effective behavioural change messages which are presented in the discussion section.

Research assistants experienced in qualitative methods conducted the interviews. Local translators were used to translate from Sidama and Oromiffa to Amharic. The interviews were tape recorded and later transcribed verbatim in Amharic. The validity of the translations was ensured by recruiting bi-lingual translators and orienting them on verbatim translations and the importance of preserving respondents’ the non-verbal expressions.

Data analysis started with a review of the Amharic transcripts. The data from individual interviews was manually summarized under each question in the interview guides. The summary data compiled under the different questions was then carefully reviewed and synthesized for write-up. Frequencies and differences in responses by respondent type and study site were noted. Responses that are especially expressive and illuminating are presented as quotes. We have followed the qualitative research review (RATS) guideline [24].

### Ethics and consent statements

The national review board- the Federal Democratic Republic of Ethiopia Ministry of Science and Technology gave ethical approval. Reference number is RDHE/87-82/2008.

As per the study protocol written consent was sought from each adult participant after reading to them about and adequately explaining the purpose of the study. Participants were told that they were free not to participate or to withdraw during any stage of the interview.

## Results

### Delivery place and care

Nearly all women interviewed gave birth at home except those few who experienced complications and delivered at a health facility. Respondents reported they often seek medical assistance for obstructed labour after waiting some time. Mothers, mothers-in-law, and other female relatives or neighbours and traditional birth attendants (TBAs) assisted most deliveries. Fathers were either absent or in the vicinity but not in the birth room or enclosure.

In preparation for delivery, most families prepare a new blade and thread to cut and tie the cord with. Grandmothers and TBAs assert that they wash their hands before and after the delivery with soap and water, a claim which may require empirical verification. For a delivery surface, people prepare *enset* (the false banana plant) leaves in the Sidama communities and sheepskin in the Oromo communities.

Some untrained TBAs said that they massage the woman’s side, abdomen or chest if they think that the baby is not descending or is coming out in the wrong position. Most of the mothers gave birth in several hours or less. A few mothers however, remained in labour from 12 to 72 hours without going to a health facility.

Grandmothers and TBAs, if present, are usually the ones who receive the baby when it is born. They receive the newborn with their bare hands unless they are trained TBAs who use gloves. Some reported to wash their hands before receiving the baby; while others do not. After the delivery of the baby, the attention of birth attendants is focused on the emergence of the placenta. The woman may be massaged if the placenta is delayed. In Gedeb Asasa, West Arsi, women are lifted upwards or put on a hollow tree trunk that is used for husking grain to help the placenta emerge. Until then, the newborn often does not receive much more attention. After the delivery, the woman is moved away from the area in which she has delivered to have her rest on a bed or the delivery surface cleaned.

### Immediate newborn care

#### Cord care

While TBAs in West Arsi are more prominent in immediate newborn care, other senior women including grandmothers, neighbours, sisters-in-law and wives of brothers-in-law also carry out this role in the other communities. After receiving the baby, birth attendants typically cut the cord with a new blade at a length ranging from a quarter to a full index finger long. Grand mothers and TBAs said that they rub the cord before cutting it to prevent blood seeping out. Some of the communities adhere to the traditional practice of allowing fathers to cut the cord in the case of male newborns. The cord is usually tied with thread, although it may not be tied at all in in some communities. Grandmothers in Aleta Chuko, Sidama said that they put ointment on the stump to help it dry up, whereas those in Liben Chikuala, East Shewa similarly apply butter in order to prevent “wind going into the baby” as well as to prevent pain and bad smells.

#### Thermal care

Although caretakers generally realize the importance of maintaining warmth for newborns, particularly protecting newborns from *berd* or the cold, babies receive little attention until the placenta is expelled. They may or may not be covered in cloth or dried. No skin to skin contact is reported and newborns are often placed slightly away from the mother at her side. Attendants are largely focused on the delivery of the placenta and the related well-being of the mother. Attendants’ orientation on the placenta is reinforced by the belief that any “harm” that comes to the placenta will directly transfer to the baby. In Sidama communities, for instance, the placenta is considered the ‘house’ or ‘blanket’ of the baby. In Gedeb Asasa, West Arsi, the father carefully buries the placenta fearing for the wellbeing of the baby if the placenta is damaged. In Liben Chikuala, East Shewa, grandmothers report breastfeeding the baby even before the placenta is expelled because they have been informed that breastfeeding helped its expulsion.

After expulsion of the placenta, most babies are bathed. The only exception to this is in Gedeb Asasa where babies are bathed with warm water and soap after the end of the first day or on their second day. In Aleta Chuko, Sidama, babies are bathed with cold water because attendants say they are too occupied by the delivery to warm some water and because they believe that “cold water makes babies grow and become fat”. Whether they are bathed or not, babies are not dried with cloth, according to most respondents. Rather the baby is simply wrapped in cloth and placed to the side of the mother or given to another caretaker.

#### Breast feeding

Early initiation of breastfeeding is variable. In most of the study communities, breastfeeding commonly commences after the placenta is expelled and the baby is bathed, although breastfeeding initiation may occur prior to bathing at times in Sidama. Mothers generally express the colostrum because it is perceived to be dirty and not good for the baby and that the subsequent breast milk is thinner and flows better. In Gedeb Asasa, West Arsi, newborns may not be breastfed before the second day due to perceptions that milk may not start to flow or that the baby is not up to it. In the meantime, newborns are given warm water with sugar:

[The first milk] is like pus or cream. We wash and massage the breasts and squeeze it out because it is not fit for the baby…. We extract it because it is dirty and so that the baby can feed easily [Grandmother, Arbe Gonna, Sidama].

### Identification and care for Low birth weight babies

Grandmothers and TBAs in most of the sites are aware of low birth babies, whereas some mothers reported that they do not know of such babies. People recognize low birth weight babies by their very thin arms and legs and how light they were when held by the hands. It is commonly believed that babies are born small if their mother was either sick or malnourished during pregnancy. ‘Those who are poor and do not live in comfort give birth to small babies’, said a twenty one year old mother in West Arsi. Others believe that low birth weight may be caused by too much work or *mitch*^a^ during pregnancy, prolonged labour, preterm birth, and a mother’s failure to drink a local herbal medicine known as *hamessa* in Sidama.

Some grandmothers and TBAs believe that babies with low birth weight are more likely to die, become sick or remain thin, and should therefore be taken to health facilities. Others believe that the babies would grow to normal weight if they receive good care such as frequent baths and breastfeeding. No other special care for low birth weight babies is mentioned.

### Post-natal care

#### Breastfeeding

In the study communities, regular and frequent breastfeeding is generally seen to be important in providing adequate nutrition to newborns and enabling them to grow. Breastfeeding is largely exclusive during the neonatal period except in the case of mothers who give babies a protective herbal mixture in the Sidama communities. One mother in Liben Chiquala reported giving water to her baby when household tasks prevented her from breastfeeding.

Most mothers say that they breastfeed babies whenever they cry and awake from sleep, or whenever they urinate in Gedeb Asasa, West Arsi. Most respondents reported that poor maternal nutrition results in inadequate breast milk. Some mothers empty a breast each time they breastfeed as they were instructed by health workers, whereas others report that they breastfed from one breast until the baby is full and then switch to the other breast when they next breastfed. Some mothers say that their breastmilk was limited because they were not eating enough themselves.

Mothers rarely give additional substances to newborns apart from Sidama mothers who had them drink the local herbal medicine, *hamesa*. Newborns that do not get *hamesa* are expected to be afflicted by a variety of illnesses.

“We give health oil and *hamessa* from the backyard to the newborn. If the baby does not get *hamessa*, he may get sick. *Hamessa* protects babies from illness. My mother washed and boiled it and gave it to me. I started having him drink it on his second day and have continued to give him half a coffee cup three times a day” [Nineteen year old mother of two children, Aleta Chuko, Sidama].

#### Thermal care

Mothers recognize the c importance of maintaining continued warmth in the neonatal period. Keeping babies clothed and covered is the most important way in which mothers attempt to keep them warm and protect them from *berd,* (the cold) – which results in a widely recognized condition in Ethiopia exhibiting symptoms such as fever, difficulty breathing, coughing, a sore throat, continuous crying, and inability to feed.

Some mothers maintain skin to skin contact with their newborns while other mothers did not. A thirty six year old mother in Aleta Chuko, Sidama, said ‘I hold my baby next to my skin to maintain her warmth, so that my heat is transferred to her’. Many mothers in Liben Chukala, East Shewa and Gedeb Asasa, West Arsi are careful to keep the baby a small distance away from them at night so as not to suffocate their babies and to prevent them from getting used to their mother’s scent and therefore cry whenever mothers had to leave them to do some household chores.

Additional ways of keeping newborns warm include the practice of exposing them to smoke daily up to the age of three months in Liben Chukala, East Shewa. The warmth that babies get is expected to fatten them. In Gedeb Asasa, West Arsi, it is common to keep a fire going on all day, which keeps up the room temperature and makes warm water available for the frequent baths that babies receive. This contrasts with the continued use of cold water to bath babies by some mothers in Sidama and East Shewa.

### Participation in newborn care

Most mothers are the primary and often the only caretakers of newborns. Some women receive assistance in caring for their newborn from their mother/mother-in-law, other in-laws, co-wives, or their own children, particularly bathing and holding the newborn. Only a few mothers report that fathers provide assistance by holding babies, washing clothes and collecting wood. Grandmothers of the newborn offer advice and guidance on various aspects of newborn care such as how to breastfeed, bath and maintain the warmth and hygiene of babies. Some fathers said that they followed up on the hygiene and warmth of their newborn.

## Discussion

The findings from this qualitative inquiry shed light on local beliefs and practices related to delivery and newborn care in rural Ethiopia, information that is of great importance for public health practitioners and policy makers. Our study findings show sub-optimal care practices are supported by long-held beliefs linked to preventing “harm” to or protecting the baby. Local perspectives which compromise maternal and newborn care include the beliefs that any harm to the placenta may put the baby at risk; cold water promotes growth of the baby; ointment on the cord stamp prevents pain and wind entering the baby; colostrum is dirty and harmful and that subsequent milk flows better; and that breastmilk does not flow well in the first day or two after birth. Perspectives, which can have a positive impact, are the importance given to frequent and regular breastfeeding and maintaining warmth, and the recognition of the association between low birth weight and malnutrition, illness and workload during pregnancy as well as pre-term birth. The study also found that grandmothers, female relatives, TBAs as well as some fathers participate in or provide advice regarding delivery and newborn care.

In order to change current practices that are not in accord with accepted standards of care [14,15,22,23,25-27], behavior change interventions that address local knowledge and perspectives related to delivery and newborn care are needed. This first requires raising awareness regarding timely and skilled attendance of delivery, clean delivery, thermal care of the newborn, hygiene, appropriate cord care, early initiation of and optimal breastfeeding and appropriate care for low birth weight. Behavioral change messages should address local concerns about delayed placenta, cord-related problems, cleanliness of colostrum, flow of breastmilk, and low birth weight. Such messages can reinforce local recognition of the importance of maternal well-being during pregnancy, newborn warmth, frequent breastfeeding and hygiene to promote optimal maternal and newborn care practices. Clearly harmful beliefs such as perceived benefits of herbal drinks, bathing newborns with cold water and exposing them to smoke should be addressed as well. Effective behaviour change messages should also target mothers, grandmothers, TBAs, female members of the extended family and fathers.

The study is not without limitations. First the study sites are limited and did not include beliefs and practices of all dominant ethnic groups that limit finding’s generalizability to other ethnic groups. The second is newborn practices are based on respondents recall and not observation. However, the study has included the major religious groups and emphasis was placed to use local terms and concepts to describe common knowledge and practices in the community.

## Conclusions

Tackling neonatal mortality is essential if Ethiopia is to achieve the millennium development goal for child mortality. Given the high levels of home delivery (90%) and lack of postnatal care in rural areas, improved newborn care practices is vital. Our findings indicate sub-optimal newborn care practices underpinned by strong cultural beliefs are prevalent in the study rural communities. Behavior change messages aimed at improving newborn practices should be informed by local perspectives related to such practices. To be fully effective, such messages should also target mothers, grandmothers, TBAs, other female family members and fathers.

## Endnote

^a^A widely accepted condition in Ethiopia in which people are affected by environmental conditions causing fever, cramps or skin sores particularly when stepping outside the house after eating or sweating.

## Competing interests

The authors declare that they have no competing interests.

## Authors’ contributions

TD has made contributions to conception and design, interpretation of data; conducted the literature review and drafted the background, discussion and conclusion sections of the manuscript. YA has made substantial contributions to conception and design; collected, analysed and synthesized the data; has drafted the methods and findings sections of the manuscript and reviewed and revised the manuscript. BM has made contributions to conception, design and interpretation of data; and reviewed and made substantial revisions to the manuscript. All authors have given final approval of the version to be published.

## Pre-publication history

The pre-publication history for this paper can be accessed here:

http://www.biomedcentral.com/1472-698X/14/17/prepub

## Supplementary Material

Additional file 1 Interview guide tools. Click here for file
