# Supplementary material for: Local understandings of care during delivery and postnatal period to inform home based package of newborn care interventions in rural Ethiopia: a qualitative study
Source: BMC Int Health Hum Rights. 2014 May 19;14:17. doi: 10.1186/1472-698X-14-17 (PMC4037276; doi:10.1186/1472-698X-14-17)
Supplement: Additional file 1 — Interview guide tools. [file 1472-698X-14-17-S1.docx]

**In-depth Interview with Traditional Birth Attendants**

**Record the following information**

Name, Age, Training, Education, Kebele, Woreda

**Child Birth**

Let us first talk about how you help deliver babies in your community.

How many babies do you deliver in a month on average?

Who or where do you help deliver babies?

When are you contacted by families to discuss your attendance at deliveries?

- Who approaches you to discuss this?
- Who makes the decision as to when you get involved?

What types of instruments do you prepare for a delivery?

- Cleaned or boiled blade
- Clean thread and cloth

Please describe in detail how you help a woman deliver a baby?

- Practices
- Who else is involved? How?
- Consultations
- Who makes decisions? What kind?

What do you think about maintaining hygiene around the delivery? Why? How?

- Hand washing before, during and after delivery
- Washing of perineal area before examinations and delivery
- Insertion of foreign material into vagina
- Cleaning of delivery surface before and during delivery
- Perceptions regarding ‘clean’ or ‘dirty’ hands, instruments and delivery surface

What danger signs and complications may be seen in a woman who is delivering a baby^[[1]](#footnote-1)^?

- Levels,
- Impact on woman and fetus/newborn
- Response
- Decision-makers/actors

When is the decision made to take the woman to a health facility? **Why?**

- Who is consulted/Decision-makers
- Influencing factors
- Constraints
- Perceptions of assistance provided by health facilities

**Newborn Health**

Immediate care of the newborn

Let us now talk about the care you give to a newborn as soon as it is delivered.

Please describe everything that you do for the baby as soon as it is born? **When**.

Inquire about the following practices including **who conducts them and why.**

- Bathing, drying, warming, placement of baby
- Breathing/crying, signs, actions taken,
- Handling of cord, instruments, boiling, substances on stump
- Breastfeeding, beliefs & practices regarding colostrum
- Additional substances given to newborn, rationale
- Conceptions and handling of placenta, impact on newborn care/feeding,
- Other practices

What consultations do you conduct on any of the above issues? **Who decides?**

Can you describe any good and bad practices for newborns that you have heard of or seen in this community?

Do some women have babies who are small/have low birth weight?

- How can you tell when a baby is small/has low birth weight?
- What is likely to happen to babies who are small/with low birth weight?
- Does a baby which is small/with low birth weight need special care? What kind?

Why do you think some newborns die just after they are born? What are the underlying causes?

What determines the survival of the newborn?

- God or spiritual forces
- Practices that enhance/compromise survival of newborn
- Signs indicative of survival possibilities of newborn
- Conditions in which survival of newborn is undesirable

Do mourning practices for newborns differ from those for older children and adults?

- Length & nature of mourning, spiritual/cultural practices
- Place and nature of burial

**Group Interview with Mother-in-laws/Grandmothers**

**Record the following information**

Name, Age, Number of children, Kebele, Woreda.

**Newborn Health**

Immediate care of the newborn

Let us first discuss the care that is given to a newborn immediately after it is born.

Who usually receives and takes care of the baby when it is born?

Please describe everything that you/she do(es) for the baby as soon as it is born? **When?**

Inquire about the following practices including **who conducts them and why**.

- Bathing, drying, warming, placement of baby
- Breathing/crying, signs, actions taken,
- Handling of cord, instruments, boiling, substances on stump
- Breastfeeding, beliefs & practices regarding colostrums
- Additional substances given to newborn, rationale
- Conceptions and handling of placenta, impact on newborn care/feeding,
- Other practices

What consultations do you conduct on any of the above issues? **Who decides?**

Do some women have babies who are small/have low birth weight?

- How can you tell when a baby is small/has low birth weight?
- What is likely to happen to babies who are small/with low birth weight?
- Does a baby which is small/with low birth weight need special care? What kind?

Why do some newborns die just after they are born? What are the underlying causes?

What determines the survival of the newborn?

- God or spiritual forces
- Practices that enhance/compromise survival of newborn
- Signs indicative of survival possibilities of newborn
- Conditions in which survival of newborn is undesirable

Do mourning practices for newborns differ from those for older children and adults? **Why?**

- Length & nature of mourning, spiritual/cultural practices
- Place and nature of burial

Neonatal care

Let us now discuss the care that is given to a newborn **in the first month of its life**.

What should a mother do for the newborn in order to ensure its survival and health?

What kind of care do you provide to your son’s/daughter’s newborn?

- What advice have you given to your daughter/daughter-in-law regarding the care of her newborn?

Can you describe any good and bad practices for newborns that you have heard of or seen in this community?

How should newborns be cared for **(why, how)**?

- Breastfeeding practices, additional/ritual substances
- Warmth
- Hygiene of and around the baby

Who decides how the newborn is cared for?

- How the baby is fed
- How the baby is kept warm
- How to maintain hygiene around the newborn

What are danger signs and complications of newborns^[[2]](#footnote-2)^?

- Local term for symptoms
- Responses
- Influencing factors
- Who is consulted/Decision-maker (Their own role?)
- Delays in care-seeking

What are sources of risk for the newborn?

What are illnesses that affect newborns?

- Symptoms
- Causes
- Responses

What are the reasons babies die in the first month after they are born?

How long should a woman and her baby stay in her home after she has given birth?

- Beliefs
- Effect on response to different newborn danger signs and illnesses
- If child is ill, who can seek care?
- Decision maker

**Group Interview with Mother-in-laws/Grandmothers**

**Record the following information**

Name, Age, Number of children, Kebele, Woreda.

**Pregnancy/Antenatal Care**

Let us first discuss the care of pregnant women in your community.

Do you have married sons or daughters who live close to you? How many?

What kinds of social activities do you maintain with your daughters or daughter-in-law?

- Visits, sharing meals, coffee ceremonies

In what ways do you and your daughters or daughters-in-law help each other?

- Farm and domestic activities, loans, child care

Do you give directions or advice to your daughter or daughter-in-law about managing their households? What kind?

- Do they listen to you?

How do you guide/advise your daughters or daughters-in-law when they are pregnant? What is your role?

Should pregnant women visit health facilities to be checked by health workers? Why? Why not?

- Availability and access
- Benefits
- Decision-maker

What are some danger signs and complications^[[3]](#footnote-3)^ that a pregnant woman may experience?

- Local terms, potential impact

What is done when a pregnant woman experiences these danger signs and complications? Why?

- Responses
- Promptness
- Influencing factors
- Who is consulted/Decision-maker

**Child Birth**

Let us now discuss the care given to women who are delivering in your community.

What preparations do families make for child birth?

Who helped your daughter/daughter-in-law deliver?

- Who else was present when she was giving birth to a baby?

Were any traditional birth attendants present?

- Training?
- Attitudes towards/benefits of trained or untrained attendants
- When and why are birth attendants requested to come to assist a delivery?
- Decision-maker on birth attendant

Please describe in detail how you (birth attendant) helped your daughter/daughter-in-law deliver a baby?

- Who else is involved? How?
- Consultations
- Who makes decisions? What kind?

What types of instruments were prepared for the delivery?

- Hygiene

What do you think about maintaining hygiene around the delivery? Why?

What is done to maintain hygiene around the delivery? Who?

- Hand washing before, during and after delivery
- Washing of perineal area before examinations and delivery
- Avoiding insertion of foreign material into vagina
- Cleaning of delivery surface before and during delivery
- Perceptions regarding ‘clean’ or ‘dirty’ hands, instruments and delivery surface

What danger signs and complications may be seen in a woman who is delivering a baby^[[4]](#footnote-4)^?

- Levels
- Impact on woman and fetus/newborn
- Response
- Decision-makers/actors

When is the decision made to take the woman to a health facility?

- Who is consulted/Decision-makers
- Influencing factors
- Perceptions of assistance provided by health facilities

**In-depth Interview with Mothers of Newborns**

**Record the following information**

Name, Age, Number of children, Education, Religion, Kebele, Woreda

**Child Birth**

Let us first discuss the care given to you when you were delivering your baby.

Please describe in detail what happened during your delivery?

- Place **(why)**, time, duration and nature of labor
- Preparation of instruments
- Maintaining hygiene

Who was present during the delivery?

- What did they do for you during the delivery?
- Was anything discussed among them?

Did you experience any problems when you were delivering your baby?

- What was done about it? Who?

What danger signs and complications may a woman experience when delivering a baby^[[5]](#footnote-5)^?

- Levels
- Impact on woman and fetus/newborn
- Response

**Newborn Health**

What has been your experience of being a mother since you had your baby?

Immediate care of the newborn

Let us now discuss the care that was given to your baby immediately after it was born.

Who received and took care of the baby when it was born?

Please describe everything that she did for the baby as soon as it was born? **Who else?**

- Drying, warming, placement of baby
- Bathing
- Breathing/crying
- Handling of cord, substances on stump
- Breastfeeding, beliefs & practices regarding colostrums
- Additional substances given to newborn, rationale

Did your baby have any problems as soon as it was born? What about other babies?

- What was done about it? Who? Any consultations/decision maker?

Do some women have babies who are small/have low birth weight?

- How can you tell when a baby is small/has low birth weight?
- What is likely to happen to babies who are small/with low birth weight?
- Does a baby which is small/with low birth weight need special care? What kind?

What determines the survival of the newborn?

Neonatal care

Let us now discuss the care that is given to a newborn in **the first month of its life.**

What do/did you do for the newborn in order to ensure its survival and health? **Why?**

- Health practices
- Spiritual practices

Who helps you take care of your baby?

- What does she/they do for your baby?
- What advice have she/they given you regarding the care of the baby?

Can you describe any good and bad practices for newborns that you have heard of or seen in this community?

How did you feed your baby?

- Exclusive breastfeeding?, additional/ritual substances, **rationale**
- Constraints - time/work, maternal nutrition, breast milk supply

Did you try to keep the newborn warm (why, how)?

- Clothing
- Contact with mother
- Bathing frequency & water/room temperature

Did you try to keep things that come into contact with the newborn clean (why, how)?

- Mode/frequency of hand washing by caretakers
- Mode of cleaning of materials

Who decides how your baby is/was cared for?

- How your baby is/was fed
- How the baby is/was kept warm
- How hygiene is/was around your baby

What are the signs of a healthy newborn?

What are sources of risk for the newborn?

What are danger signs and complications of newborns^[[6]](#footnote-6)^? Did your baby have them?

- Local term for symptoms
- Responses – expected and actual
- Influencing factors
- Who is/was consulted/Decision maker
- Delays in care seeking

What are illnesses that affect newborns?

- Symptoms
- Severity
- Causes
- Responses

What are the reasons babies die in the first month after they are born?

How long did you and your baby have to stay in your home after you gave birth?

- Beliefs
- Effect on response to different newborn danger signs and illnesses
- If child was ill, who sought care?
- Decision maker

What type of nutrition did you have in the first month after you gave birth?

- Type and relative amount
- Beliefs
- Impact on neonatal health

How much work did you do in the first month after you gave birth?

- Type and relative amount
- Assistants
- Beliefs
- Impact on newborn health

**Pregnancy/Antenatal Care**

We shall now discuss the care of pregnant women in your community.

What should a pregnant woman do to maintain her health and that of the newborn? Why?

- Beneficial/harmful foods, quantity
- Workload, activity type

How did you find out you were pregnant?

- Who did you tell when you first found out you were pregnant? When?
- What was discussed or done with regard to the pregnancy?

Should pregnant women visit health facilities to be checked by health workers? Why not?

- Availability and access – distance, cost, service hours
- Attitudes towards health staff - gender
- Benefits, procedures conducted
- Quality of service
- Desirable and actual frequency of visits
- Social/male support for ANC visits
- Decision-maker

What are some danger signs and complications^[[7]](#footnote-7)^ that a pregnant woman may experience?

- Local terms, potential impact

Which danger signs and complications did you experience?

What should be (was) done for a (you) pregnant woman who experiences these danger signs and complications? Why?

- Responses
- Promptness
- Influencing factors
- Who is consulted/Decision-maker

What did you do to prepare for giving birth?

- Who helped you prepare to give birth? How?
- Who did you discuss the birth with? What?
- Were emergency preparations discussed? What?
- Decision maker

**In-depth Interview with Fathers of Newborns**

**Record the following information**

Name, Age, Number of children, Education, Religion, Kebele, Woreda

**Newborn Health**

Neonatal care

Let us now discuss the care that is given to a newborn **in the first month of its life.**

What has been your experience of being a father since you had your baby?

What do/did you do for the newborn and/or mother in order to ensure your baby’s survival and health?

- Health practices
- Spiritual practices
- Economic support
- Other support: doing woman’s chores, ensuring mother/baby get rest, etc.
- Decision-making on health care
- Who did you consult?

What are sources of risk for the newborn?

How should newborns be cared for **(why, how)**?

- Breastfeeding practices, additional/ritual substances
- Warmth
- Hygiene of and around the baby

Who makes the decisions regarding the following aspects of the care of your newborn?

- What happens to the baby immediately after birth
- How the umbilical cord is cut and treated
- When and how often the baby is bathed
- How the baby is kept warm
- When the baby is breastfed
- Maintaining hygiene around the baby

Are/were you involved in each of these newborn care practices? How?

Would you like to be more involved in decisions about how your newborn baby is cared for?

Would your wife (or the decision-maker(s) mentioned above) take advice that you gave her about the newborn?

What are the signs of a healthy newborn?

What are danger signs and complications of newborns^[[8]](#footnote-8)^?

- Local term for symptoms
- Responses
- Influencing factors
- Who is consulted/Decision-maker

How long did your wife and baby stay in the house after she gave birth?

- Beliefs
- Influencing factors
- Effect on response to different newborn danger signs and illnesses

**Child Birth**

Let us now discuss a few things about your wife’s delivery.

Were you present when your wife was giving birth to a baby? Why not?

- Role

Who helped your wife to deliver?

- Decision-maker on birth attendant

What danger signs and complications may be seen in a woman who is delivering a baby^[[9]](#footnote-9)^?

- Impact on woman and fetus/newborn

When would you have taken your wife to a health facility to deliver?

- Level of complication
- Consultation with TBA and other women
- Decision Makers

**Pregnancy/Antenatal Care**

We shall now discuss the care of pregnant women in your community.

What should a pregnant woman do to maintain her health and that of the newborn? Why?

- Beneficial/harmful foods, quantity
- Workload, activity type

Should pregnant women visit health facilities to be checked by health workers? Why not?

- Benefits/procedures
- Desirable frequency of visits
- Decision-maker

What are some danger signs and complications^[[10]](#footnote-10)^ that a pregnant woman may experience?

- Potential impact

What should be done for a pregnant woman who experiences these danger signs and complications? Why?

- Responses
- Promptness
- Influencing factors
- Who is consulted/Decision-maker

How were you consulted about the delivery and care of the newborn?

- By whom?
- Level of discussion with wife on what was to happen
- Level of discussion between husband and his mother about what was to happen.

What did you do to prepare for your wife’s delivery? Why not?

- Assuring emergency transportation to a health facility
- Setting aside sufficient money for emergency transportation & medical care

1. Bleeding, fever, convulsions (fits), loss of consciousness, prolonged labor > 12 hours, preterm labor, prolapsed cord or noncephalic presenting part, meconium discharge during labor, placenta not expelled one hour after birth. [↑](#footnote-ref-1)
2. Inability to feed adequately or suck, weak/abnormal cry or cessation of crying, lethargy or loss of consciousness, redness of umbilicus and abdomen, pus from the umbilicus, localized skin infection, discharge from or redness of the eyes, persistent vomiting and/or abdominal distention, difficulty breathing, fast breathing, fever or cold body temperature, convulsions or fits, yellow discoloration (jaundice) of the skin. [↑](#footnote-ref-2)
3. Priority danger signs - vaginal bleeding, fever, convulsions (fits), loss of consciousness (fits), severe headaches with or without dizziness, difficulty breathing, contractions/labor pains or water breaking before 37 weeks gestation. [↑](#footnote-ref-3)
4. Bleeding, fever, convulsions (fits), loss of consciousness, prolonged labor > 12 hours, preterm labor, prolapsed cord or noncephalic presenting part, meconium discharge during labor [↑](#footnote-ref-4)
5. Bleeding, fever, convulsions (fits), loss of consciousness, prolonged labor > 12 hours, preterm labor, prolapsed cord or noncephalic presenting part, meconium discharge during labor [↑](#footnote-ref-5)
6. Inability to feed adequately or suck, weak/abnormal cry or cessation of crying, lethargy or loss of consciousness, redness of umbilicus and abdomen, pus from the umbilicus, localized skin infection, discharge from or redness of the eyes, persistent vomiting and/or abdominal distention, difficulty breathing, fast breathing, fever or cold body temperature, convulsions or fits, yellow discoloration (jaundice) of the skin. [↑](#footnote-ref-6)
7. Priority danger signs - vaginal bleeding, fever, convulsions (fits), loss of consciousness (fits), severe headaches with or without dizziness, difficulty breathing, contractions/labor pains or water breaking before 37 weeks gestation. [↑](#footnote-ref-7)
8. Inability to feed adequately or suck, weak/abnormal cry or cessation of crying, lethargy or loss of consciousness, redness of umbilicus and abdomen, pus from the umbilicus, localized skin infection, discharge from or redness of the eyes, persistent vomiting and/or abdominal distention, difficulty breathing, fast breathing, fever or cold body temperature, convulsions or fits, yellow discoloration (jaundice) of the skin. [↑](#footnote-ref-8)
9. Bleeding, fever, convulsions (fits), loss of consciousness, prolonged labor > 12 hours, preterm labor, prolapsed cord or noncephalic presenting part, meconium discharge during labor [↑](#footnote-ref-9)
10. Priority danger signs - vaginal bleeding, fever, convulsions (fits), loss of consciousness (fits), severe headaches with or without dizziness, difficulty breathing, contractions/labor pains or water breaking before 37 weeks gestation. [↑](#footnote-ref-10)
